# Clinical results of active surveillance for extra‐abdominal desmoid‐type fibromatosis

**DOI:** 10.1002/cam4.5329

**Published:** 2022-10-09

**Authors:** Tomohisa Sakai, Yoshihiro Nishida, Kan Ito, Kunihiro Ikuta, Hiroshi Urakawa, Hiroshi Koike, Shiro Imagama

**Affiliations:** ^1^ Department of Orthopedic Surgery Nagoya University Graduate School of Medicine Nagoya Japan; ^2^ Rare Cancer Center Nagoya University Hospital Nagoya Japan; ^3^ Department of Rehabilitation Nagoya University Hospital Nagoya Japan

**Keywords:** active surveillance, active treatment, CTNNB1, desmoid, neck

## Abstract

**Background:**

The treatment of choice for desmoid‐type fibromatosis (DF) has been changed to active surveillance (AS). However, few studies have reported clinical outcomes of AS modality in Asian countries. This study aimed to clarify the significance of AS as a DF treatment modality.

**Methods:**

A total of 168 lesions from 162 patients with extra‐abdominal DF were included. The mean age at diagnosis was 39 years (1–88 years), and the median maximum tumor diameter at the first visit was 64.1 mm (13.2–255.8 mm). The clinical outcomes of AS and the risk factors requiring active treatment (AT) (defined as an event) from AS modality were investigated.

**Results:**

Of the 168 lesions, 94 (56%) were able to continue AS, with a 5‐year event‐free survival of 54.8%. Of the 68 lesions with PD, 21 (30.9%) lesions were able to continue AS. Neck location (*p* = 0.043) and *CTNNB1* S45F mutation (*p* = 0.003) were significantly associated with the transition to AT, and S45F mutation was a significant factor associated with the transition to AT by multivariate analysis (hazard ratio: 1.96, *p* = 0.048). AT outcomes after AS were evaluable in 65 lesions, and 49 (75%) lesions did not require a transition to a second AT.

**Conclusions:**

AS was revealed as an effective treatment modality. The transition to AT needs to be considered for neck location and *CTNNB1* S45F mutation DF. Good results can be obtained by selecting a treatment method that considers the tumor location even in cases that require intervention.

## Graphical abstract

Active surveillance was prospectively performed on 168 cases of extra‐abdominal desmoid and could be continued in 94 (56%) cases. Desmoid with CTNNB1 S45F mutation and neck location were associated with active treatment intervention. Active treatment outcomes after active surveillance were evaluable in 65 lesions, and 49 (75%) lesions did not require a transition to a second active treatment.

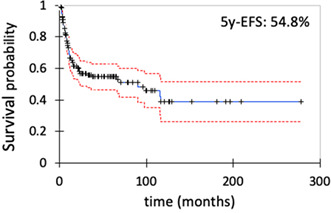

## 1 INTRODUCTION

Desmoid‐type fibromatosis (DF) is a (myo‐) fibroblastic soft tissue tumor that is classified as an intermediate malignancy according to the World Health Organization classification.^1^ DF is highly locally invasive and has a high postoperative recurrence rate. It does not cause distant metastasis like soft tissue sarcomas. Additionally, it may spontaneously regress or disappear without any treatment in some patients, thus it has been named an “enigmatic” lesion.^2^ DF is a rare tumor that occurs in less than four people per million people annually.^3^, ^4^ Approximately, 90% of DF are sporadic and often carry mutations in the hot spot of the β‐catenin gene (*CTNNB1*).^5^ DF occurs in any part of the body, but particularly in the limbs and trunk. The remaining 10% of DF occurs in patients with familial adenomatous polyposis (FAP), which mutated in the adenomatous polyposis coli gene, and those with FAP mainly in the abdominal cavity.^6^

The standard of treatment modality for DF has been surgical resection, generally with a wide surgical margin, similar to those for soft tissue sarcomas.^7^, ^8^ However, even after extensive resection with significant loss of function, a high recurrence rate of 20%–40% has been reported.^9^, ^10^ Systemic therapy, including chemotherapy, has been reported as a possible treatment alternative other than surgical resection; however, the activities of daily living (ADL) disturbance due to adverse events by the treatment is a considerable problem.^11^, ^12^ Considering the surgical and systemic treatment results, in addition to the occasional spontaneous regression and inability of distant metastasis in DF, careful follow‐up (active surveillance: AS) has been conducted on patients with DF with slight or moderate symptoms. Active treatment (AT), including surgery and/or systemic treatments, will be considered in cases of tumor growth that causes severe pain, functional impairment, and life‐threatening situations after the treatment modality of AS.^13^, ^14^, ^15^ Several studies have reported clinical outcomes for AS in western countries; however, few have reported the significant factors associated with the transition from AS to AT.^13^, ^14^, ^15^ Moreover, the clinical outcome of AS for DF has not been reported yet in Asian countries, including Japan.

This study aimed to clarify the clinical outcomes of AS for patients with DF and identify the leading risk factors of AT after AS at our institution. Additionally, AT methods and their outcomes after AS modality is also investigated.

## 2 MATERIALS AND METHODS

Since 2000, 204 patients were diagnosed with DF and treated at our institution. Among them, patients who received AS modality and followed up and evaluated for >6 months or until AT intervention were included in this study. Both primary and recurrent patients were included. Patients with intraabdominal location, with a history of chemotherapy, were excluded. Patients with a history of use of COX‐2 inhibitors, tranilast, and tamoxifen were included because these drugs have been reported to have no evidence of effects for DF.^15^ Finally, a total of 162 patients (168 lesions) were subjected to the analyses, including a patient with FAP.

Among 168 lesions, 56 arose in males and 112 in females and 140 were primary and 28 were recurrent. Additionally, 20 lesions were found in the neck, 50 in the limbs, 45 in the abdominal walls, 47 in other trunks, and 6 in the retroperitoneal spaces. The average age at diagnosis was 39 years (1–88 years), and the median maximum tumor diameter was 64.1 mm (13.2–255.8 mm). A history of medication, without clear evidence of effects^15^ during the follow‐up, was determined in 126 lesions. Multiple medications were given to 20 lesions. A COX‐2 inhibitor was used for 116 lesions, tranilast for 26 lesions,^16^ and tamoxifen for 3 lesions. No patients in this study cohort received radiotherapy. Mutation analysis was performed for *CTNNB1* on 154 lesions using the Sanger sequencing.^17^

DF lesions were generally evaluated with magnetic resonance imaging (MRI) every 3–6 months. If MRI could not be performed due to patients' condition, lesions were evaluated using computed tomography. Two lesions in one patient were independently evaluated. Response Evaluation Criteria in Solid Tumors (RECIST) were used to evaluate the efficacy of AS in patients with DF to assess the changes in tumor burden.^18^ Trained physicians (Y.N. and K.I.), who are blinded to clinical information, evaluated the greatest tumor diameter and changes according to RECIST 1.1. AT intervention is determined after careful consultation with the patient, considering tumor growth on imaging, pain, and impairment of the patient's ADL/QOL due to the tumor. AS is continued if the tumor exhibits PD, however, does not cause deterioration in ADL/QOL, such as worsening limited range of motion or worsening pain. Conversely, if the tumor develops near a joint and causes a limitation in joint range of motion, or if the tumor develops near a vital organ or neurovascular bundle and there is concern about organ damage, AT is carried out in cases not showing PD.

Comparisons of categorical variables between groups were performed using the chi‐square test separated by the median. The start date was defined as the date of diagnosis, which was identical to AS start date, to determine the event‐free survival (EFS). The event was defined as AT intervention. EFS was determined using the Kaplan–Meier method, and comparisons between groups were performed using the log‐rank test. Significant factors associated with the EFS (at *p* < 0.05) in the univariate analysis, and age at diagnosis and tumor size, which have been reported as poor prognosticators in DF,^5^, ^9^ were subsequently analyzed with multivariate Cox proportional hazard regression analyses of covariates. All statistical analyses were performed using IBM Statistical Package for the Social Sciences version 28. A *p*‐value of <0.05 was considered significant.

This study was approved by the Institutional Review Board (approved number: 1322). Informed consent was partly obtained from patients, and the need for informed consent was partly waived because of the retrospective design of the study based on anonymous data. This study was conducted following the principles set out in the Declaration of Helsinki.

## 3 RESULTS

**FIGURE 1:**
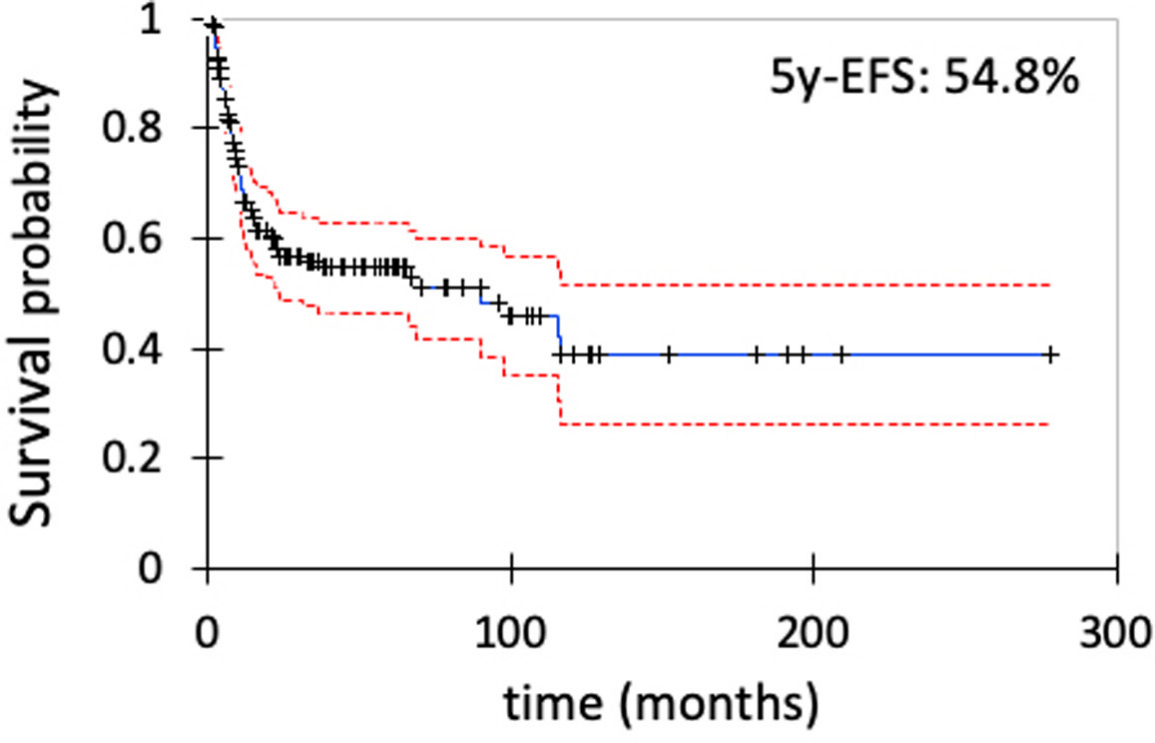
Kaplan–Maier curve for event‐free survival with active surveillance policy in the entire cohort (*n* = 168 lesions).

The characteristics of 168 lesions are shown in Table 1. *CTNNB1* mutation status (*n* = 154) was T41A in 91 lesions, S45F in 17, S45P in 6, T41I in 5, H36P in 1, and wild‐type in 34. Image evaluation using RECIST could be achieved at the final examination or the start of AT intervention for 156 lesions. The status was complete response (CR) in 7, partial response (PR) in 17, stable disease (SD) in 64, and progressive disease (PD) in 68 lesions, with an average time and mean time to PD of 15 months and 9 months retrospectively (0–64 months). The 5‐year PD‐free survival was 50.0% (Figure S1). Of the 168 lesions, 94 (56%) were able to continue AS modality, with a 5‐year EFS of 54.8%. A transition from AS to AT was required in 74 (44%) lesions, with an average and mean time to the intervention of 15 months and 8 months (1–90 months), respectively (Figure 1). Of the 94 lesions for which AS could be continued, response to AS could be evaluated in 89 lesions by RECIST, and the outcomes were CR in 7, PR in 17, SD in 44, and PD in 21. Of the 74 lesions that required AT, 67 cases could be evaluated by RECIST, and the outcomes were SD in 20 and PD in 47. Of these 20 lesions with SD, 18 (90%) showed tumor growth that did not reach PD. PD status by RECIST showed a significant association with the transition to AT (*p* < 0.0001, chi‐square test). Age, gender, tumor diameter at the first visit, tumor location, recurrent lesions*, CTNNB1* S45F mutation, and history of drug administration did not show any significant association with PD (Table 2). The reasons for the transition to AT in patients with SD (*n* = 20) were to prevent the occurrence of functional impairment in 9 cases, to reduce pain in 6 cases, to regulate tumor growth (evaluation: SD) in 4 cases, and patient wishes in 1 case.

**TABLE 1:** Patient characteristic.

Characteristic	N	%
Gender		
Female	112	67
Male	56	33
Primary/Recurrent		
Primary	140	83
Recurrent	28	17
Mean age, years (range)	39.1 (1–88)	
Mean size, mm (range)	74.7 (13.2–255.8)	
Site		
Extremities	50	30
Abdominal wall	45	27
Other trunk	47	28
Retroperitoneal	6	4
Neck	20	12
*CTNNB1* mutation (*n* = 154)		
T41A	91	59
T41I	5	3
S45F	17	11
S45P	6	4
H36P	1	1
Wild type	34	22
Drug administration		
COX‐2 inhibitor	116	69
Tranilast	26	15
Tamoxifen	3	2
None	42	25
RECIST (*n* = 156)		
CR	7	4
PR	17	11
SD	64	41
PD	68	44

Abbreviations: CR, complete remission; PD, progressive disease; PR, partial remission; SD, stable disease.

**TABLE 2:** Univariate analysis of factors associated with tumor progression.

Factor	Non‐PD	PD	*p* value
Gender			
Female	55	50	0.14
Male	33	18	
Age			
≤33	31	32	0.14
>33	57	36	
Size			
<64 mm	39	38	0.15
>64 mm	49	30	
Primary/Recurrent			
Primary	75	55	0.47
Recurrent	13	13	
Site (Neck/Other)			
Neck	8	9	0.41
Other	80	59	
Site (Extremity/Other)			
Extremity	22	25	0.11
Other	66	43	
*CTNNB1* mutation (*n* = 143)			
S45F	6	9	0.19
Other	74	54	
Drug administration			
Presence	63	54	0.26
Absence	25	14	
Conversion to AT			
Presence	20	47	<0.0001
Absence	68	21	

Abbreviations: AT, active treatment; PD, progressive disease.

**FIGURE 2:**
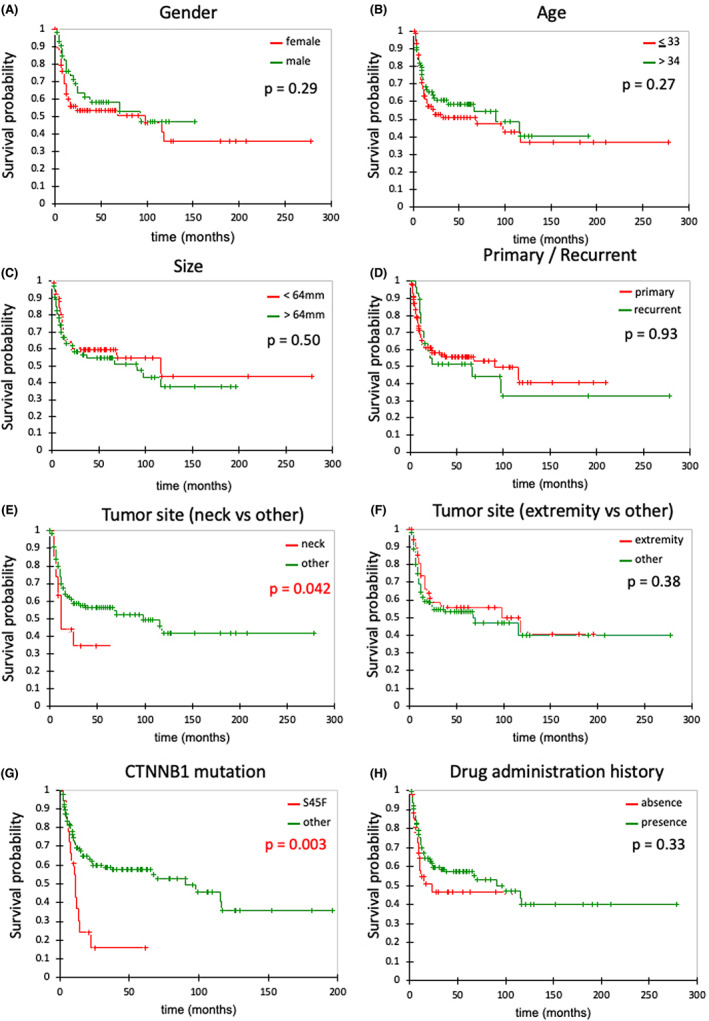
Event‐free survival curve for various factors. Kaplan–Meier curve for (A) gender, (B) age, (C) tumor size, (D) primary or recurrent tumor, (E) tumor site (neck vs. other), (F) tumor site (extremity vs. other), (G) *CTNNB1* mutation status (S45F vs. other), and (H) drug administration history.

Neck location (*p* = 0.042) and *CTNNB1* S45F mutation (*p* = 0.003) were significantly associated with poor EFS. Contrarily, age (*p* = 0.27), gender (*p* = 0.29), tumor diameter at first visit (*p* = 0.50), limb development (*p* = 0.38), recurrent lesions (*p* = 0.93), and drug administration history (*p* = 0.33) did not show any significant association with EFS (Figure 2) (Table 3). Tables S1 and S2 show the relationship between the tumor site, *CTNNB1* mutation status, and PD versus non‐PD, AS continuation versus AT transition, respectively. The COX‐hazard multivariate analysis revealed a significant relationship between neck location and poor EFS (hazard ratio: 1.96, 95% confidence interval: 1.01–3.80, *p* = 0.048).

**TABLE 3:** Univariate and multivariate analyses of factors associated with event‐free survival.

Factor	*n*	Events	Univariate HR (95% CI)	Univariate p value	Multivariate HR (95% CI)	Multivariate *p* value
Gender						
Female	113	53	1.3 (0.8–2.2)	0.29	NA	
Male	55	21	Ref			
Age						
≤33	93	39	1.3 (0.8–2.0)	0.27	1.13 (0.69–1.83)	0.63
>33	75	34	Ref		Ref	
Size (*n* = 162)						
<64 mm	79	32	Ref	0.50	Ref	0.26
>64 mm	83	39	1.2 (0.8–2.0)		1.29 (0.81–2.16)	
Primary/Recurrent						
Primary	141	60	Ref	0.93	NA	
Recurrent	27	14	0.98 (0.6–1.8)			
Site (Neck/Other)						
Neck	20	12	1.9 (1.0–3.5)	0.042	1.80 (0.86–3.75)	0.12
Other	148	62	Ref		Ref	
Site (Extremity/Other)						
Extremity	50	21	0.80 (0.5–1.3)	0.38	NA	
Other	118	53	Ref			
*CTNNB1* mutation (*n* = 154)						
S45F	17	13	2.4 (1.3–4.5)	0.003	1.96 (1.01–3.80)	0.048
Other	137	59	Ref		Ref	
Drug administration						
Presence	124	54	0.8 (0.5–1.3)	0.33	NA	
Absence	44	19	Ref			

Abbreviations: NA, Not assessed.

Treatment details for 74 lesions that received AT include low‐dose chemotherapy with methotrexate and vinblastine (MTX + VBL) in 46 lesions, surgery in 24, pazopanib in 3, and oral administration of methotrexate in 1. AT for abdominal wall lesions include surgery in 14 of 22 (64%), which was significantly more selected compared to other tumor sites (*p* = 0.05, chi‐square test) (Table 4).

**TABLE 4:** Active treatment modality and tumor site.

	Tumor site					*p* value
	EX	AW	OT	RP	Neck	
Treatment modality						0.05
MTX + VBL (*n* = 46)	14	8	12	2	10	
Pazopanib (*n* = 3)	1	0	1	0	1	
Surgery (*n* = 24)	5	14	5	0	0	
Oral MTX (*n* = 1)	1	0	0	0	0	

Abbreviations: AW, Abdominal wall; EX, Extremity; MTX, Methotrexate; OT, Other trunk; RP, Retroperitoneal; VBL, Vinblastine.

AT outcomes were evaluable in 65 of 74 lesions (MTX + VBL: 44, surgery: 20, and pazopanib: 1). Of the 44 lesions with MTX + VBL treatment, 33 did not require another AT intervention because tumor shrinkage or symptom relief was obtained, whereas 11 had to shift to another AT due to tumor progression or worsening of pain or functional impairment, or adverse event of MTX + VBL. As a treatment following MTX + VBL, surgery was selected in 5 cases, pazopanib in 4 cases, and adriamycin‐based chemotherapy in 2 cases. Of the 20 evaluable lesions with surgery as AT, recurrences were seen in 5 (25%), with tumor locations of 1 in the abdominal wall, 2 in the extremity, and 2 in the other trunks. One lesion with pazopanib as AT had tumor regression and did not require the next treatment. Overall, 49 of the 65 (75%) lesions that received AT intervention after AS did not require the next AT.

## 4 DISCUSSION

The treatment modalities for DF have changed from surgery with a wide surgical margin to AS.^15^ Our institution has discontinued the surgical treatment as an initial treatment since 2003 in most cases, and the AS policy is being applied with or without COX‐2 inhibitor.^19^ Several clinical results on AS for desmoids have been reported from western countries (Table 5). Studies were reported from Italy, France,^20^, ^21^, ^22^, ^24^ the United States,^28^ the Netherlands,^25^ the United Kingdom,^27^ Canada,^23^, ^26^ Poland,^30^ and European Musculo‐Skeletal Oncology Society^29^: however, no study has reported on clinical outcomes of AS from Asian countries, including Japan. Making a strict comparison between previous studies and the present one is difficult; however, clinical outcomes of past reports indicated that tumor progression was observed from 14% to 54% with AS policy, and the transition rate to AT was from 18% to 58%. These results are comparable to those of the present study, PD was 46% and the transition rate to AT was 44%. Additionally, previous studies that investigated the outcomes of AS revealed that many patients were treated with upfront surgery or chemotherapy followed by AS policy. Penel et al. reported 388 patients with AS policy, of them 359 had initial surgical treatment.^24^ Van Houdt et al. reported 168 patients with AS; however, their study had 584 patients with DF, and 416 patients received immediate surgical or systemic treatment.^27^ Cassidy's study from Memorial Sloan Kettering Cancer Center reported the clinical outcomes of 72 patients with AS, whereas 88 patients received immediate intervention mainly with surgery.^28^ Hence, these studies did not consist of cohorts that strictly adhered to the initial AS policy. Compared with these previous reports, the rate of adherence to the AS policy was high in the cohort of the present study (168 in 204 lesions; 82%), and the results are expected to show real clinical outcomes of AS for DF.

**TABLE 5:** Studies about active surveillance in desmoid‐type fibromatosis.

Reference	*N*	Tumor site	PD	Shift to AT	Risk factor
Fiore, 2009^20^	83	Extremity 27 Trunk 5 Head and neck 3 Abdominal wall 33 Thoracic wall 9 Intra‐abdominal 6	29 (35%)	23 (28%)	NS
Bonvalot, 2013^21^	102	Abdominal wall 102	NS	37 (36%)	Size > 3.5 cm
Colombo, 2015^22^	70	Intra‐abdominal 10 Extremity/girdle 26 Head/neck 2 Trunk 32	28 (40%)	28 (40%)	NS
Burtenshaw, 2016^23^	120	Abdominal wall 63 Intra‐abdominal 49 Both 8 (all FAP patients)	NS	53 (44%)	NS
Penel, 2017^24^	388	NS	117 (30%)	71 (18%)	NS
Van Broekhoven, 2018^25^	37	Head/neck 3 Thorax/back 13 Abdominal wall 13 Extremity 4	5 (14%)	15 (41%)	NS
Turner, 2019^26^	50	Head/neck 3 Upper extremity 4 Lower Extremity 14 Abdominal wall 14 Intra‐abdominal 3 Chest wall 6 Breast 2 Limb‐girdle 2 Other 2	21 (42%)	19 (38%)	NS
Van Houdt, 2019^27^	168	Lower extremity 20 Upper extremity 31 Abdominal wall 61 Intra‐abdominal 15 Chest wall 30 Other 11	60 (36%)	78 (46%)	Size ≥ 7 cm Pain RECIST PD
Cassidy, 2020^28^	72	Abdominal wall 19 Chest wall 5 Intra‐abdominal 21 Extremity 21 Other 6	NS	42 (58%)	Size >5 cm Paraspinal/frank location
Cuomo, 2021^29^	70	Upper extremity 20 Lower extremity 22 Pelvic girdle 26 Shoulder girdle 31	38 (54%)	NS	Pain Shoulder location
Soboczuk, 2021^30^	139	Abdominal wall 46 Chest wall 40 Upper extremity 10 Lower extremity 25 Intra‐abdominal 15	60 (43%)	41 (29%)	NS
This study	168	Neck 20 Extremity 50 Abdominal wall 45 Other trunk 47 Retroperitoneal 6	71/156 (46%)	74/168 (44%)	Neck location *CTNNB1* S45F RECIST PD

Abbreviations: AT, active treatment; FAP, familial adenomatous polyposis; NS, not specified; PD, progressive disease.

A few literature has reported risk factors for patients with AS to transfer to AT in DF. Bonvalot et al. reported that DF larger than 3.5 cm was significantly associated with the intervention.^21^ Van Houdt et al. and Cassidy et al. also reported a significant correlation with tumor size with AT, larger than 7 and 5 cm, respectively^27^, ^28^ . However, Cassidy's study could not reveal the relationship between tumor size and progression‐free survival. No association was found between the tumor size and AT transition in our study. The decision to choose AT for tumor size may differ between institutions or ethnic groups. Neck location was significantly associated with AT in the present study using univariate analysis. Interestingly, cases of neck location were not significantly associated with tumor progression. DF of neck location is likely to be in contact with important organs, such as the trachea, cervical/brachial plexus, and carotid blood vessels, thus physicians and patients may choose AT earlier than those in other locations before the tumor may harm these organs. Previous studies that investigated the risk factors of patients with AS to AT^27^, ^28^, ^29^ did not extract the neck location as a significant factor. In these three research reports, the neck is not classified as an independent site, which might be the difference from our study results. In DF, the neck is different from other locations because of the proximity to important organs, thus it had better be classified as an independent site.

The present study revealed the *CTNNB1* S45F mutation as a significant poor prognostic factor for EFS using multivariate analysis. Colombo et al. also reported that DF with the S45F mutation tended to have shorter treatment‐free survival (*p* = 0.06).^31^ The *CTNNB1* S45F mutation has been previously reported as a risk factor for recurrence after surgical treatment^5^, ^32^, ^33^, ^34^ and associated with tumor progression in patients with oral COX‐2 inhibitor treatment.^17^ Contrarily, drug treatment with considerable side effects, including anticancer drugs and molecular targeted drugs, has been reported to control DF with any type of *CTNNB1* mutation.^35^, ^36^ Altogether, DF that cannot be controlled by AS can be suppressed by anticancer drugs or molecular target drugs even if it is S45F. Tumor diameter, gender, recurrent cases, limb location, and juvenile development have been reported as risk factors for recurrence regarding surgery.^4^, ^34^, ^37^, ^38^ However, none of them were significantly associated with EFS in the present study that investigated AS policy. These might indicate that the AS policy can be selected without considering the influence of these factors.

DF often causes pain, and studies by van Houde and Cuomo et al. reported that pain was a risk factor for AT.^27^, ^29^ Reportedly, pain is associated with 80% of the patients,^39^ thus evaluating the degree and type of pain in the future with the evaluation of numerical rating scale and/or neuropathic pain scale to evaluate the benefit of AS policy.

AS is recommended for DF with non‐to‐moderate symptoms. Effective AT should be considered in cases with worsened symptoms, impaired ADLs, and tumors getting closer to an important organ. Surgical resection, which has been the mainstay of the treatment, has a high recurrence rate,^9^, ^10^, ^38^ and the functional impairment may be worsened by surgery and/or recurrent tumors. The consensus statement recommends surgical treatment for DF with abdominal wall development,^15^ and previous studies demonstrated the favorable outcomes of surgery for abdominal wall DF.^21^, ^40^, ^41^ Effective medical treatments are recommended, including low‐dose chemotherapy with MTX + VBL,^12^, ^35^ or multikinase inhibitor therapy with sorafenib^42^ or pazopanib, for other locations.^43^ In the present study, the most used AT was MTX + VBL, whereas the surgical intervention was significantly more performed on the abdominal wall DF compared to other sites. Reportedly, abdominal wall DF has a lower postoperative recurrence rate than other sites,^21^, ^39^, ^40^, ^41^ and surgery is also considered for DF intervention in cases with abdominal wall involvement.

This study has several limitations. First, as with any previous studies, the criteria for performing AT have been determined by close consultation with the individual physicians and patients. The criteria will be different from past reports. Second, risk factors for AT have been identified; however, factors that influence the time to AT have not been investigated. Studies will be needed to identify the factors involved in the time to AT. Third, patient‐reported outcomes were not investigated in the present study, which might be the most important for AS policy. In some patients, pain and disability status prior to initiation of AS were not quantified. Since these factors influence subsequent AT interventions, it is important to assess pre‐AS status in the future. Additionally, because *CTNNB1* mutation analysis is performed by the Sanger sequencing, false‐negative cases may be included in the cohort. Whole‐exome sequencing may increase *CTNNB1* mutation‐positive cases.^44^ However, the present study contains a large number of cases of extra‐abdominal DF, and many cases have *CTNNB1* mutation analyses.

In conclusion of the present study, AS was prospectively performed on 168 cases of extra‐abdominal DF and could be continued in 94 (56%) cases. Given that initial AT is rarely performed in the era of this cohort, the results show the clinical outcomes of actual AS policy. DF with *CTNNB1* S45F mutation and neck location were associated with AT intervention. The timing and modality of AT intervention require comprehensive judgment, based on the tumor location, involved lesion function, and patient satisfaction, including pain.

## AUTHOR CONTRIBUTIONS

**Tomohisa Sakai:** Data curation (supporting); formal analysis (lead); investigation (lead); methodology (lead); resources (supporting); writing – original draft (lead). **Yoshihiro Nishida:** Conceptualization (lead); data curation (supporting); formal analysis (supporting); funding acquisition (lead); investigation (supporting); methodology (supporting); project administration (lead); validation (supporting); visualization (lead); writing – original draft (supporting); writing – review and editing (lead). **Kan Ito:** Data curation (supporting); investigation (supporting); methodology (supporting); resources (supporting); writing – review and editing (supporting). **Kunihiro Ikuta:** Data curation (supporting); methodology (supporting); resources (supporting); writing – review and editing (supporting). **Hiroshi Urakawa:** Data curation (supporting); methodology (supporting); resources (supporting); validation (supporting). **Hiroshi Koike:** Data curation (supporting); investigation (supporting); resources (supporting); writing – review and editing (supporting). **Shiro Imagama:** Funding acquisition (supporting); supervision (supporting); writing – review and editing (supporting).

## FUNDING INFORMATION

This work was supported part by the Ministry of Health, Labor and Welfare of Japan, the Ministry of Education, Culture, Sports, Science, and Technology of Japan [Grant‐in‐Aid 17H01585 for Scientific Research (A)], and the National Cancer Center Research and Development Fund (29‐A‐3).

## CONFLICTS OF INTEREST

All authors declare that they have no conflict of interest to declare.

## ETHICS STATEMENT

This study was approved by the Institutional Review Board (approved number: 1322). Informed consent was partly obtained from patients, and the need for informed consent was partly waived because of the retrospective design of the study based on anonymous data. This study was conducted following the principles set out in the Declaration of Helsinki.

## Supporting information


Figure S1

Table S1

Table S2

## Data Availability

Data available on request from the authors.
