# Supplementary figures and images for: Clinical results of active surveillance for extra‐abdominal desmoid‐type fibromatosis
Source: Cancer Med. 2022 Oct 9;12(5):5245–54. doi: 10.1002/cam4.5329 (PMC10028109; doi:10.1002/cam4.5329)

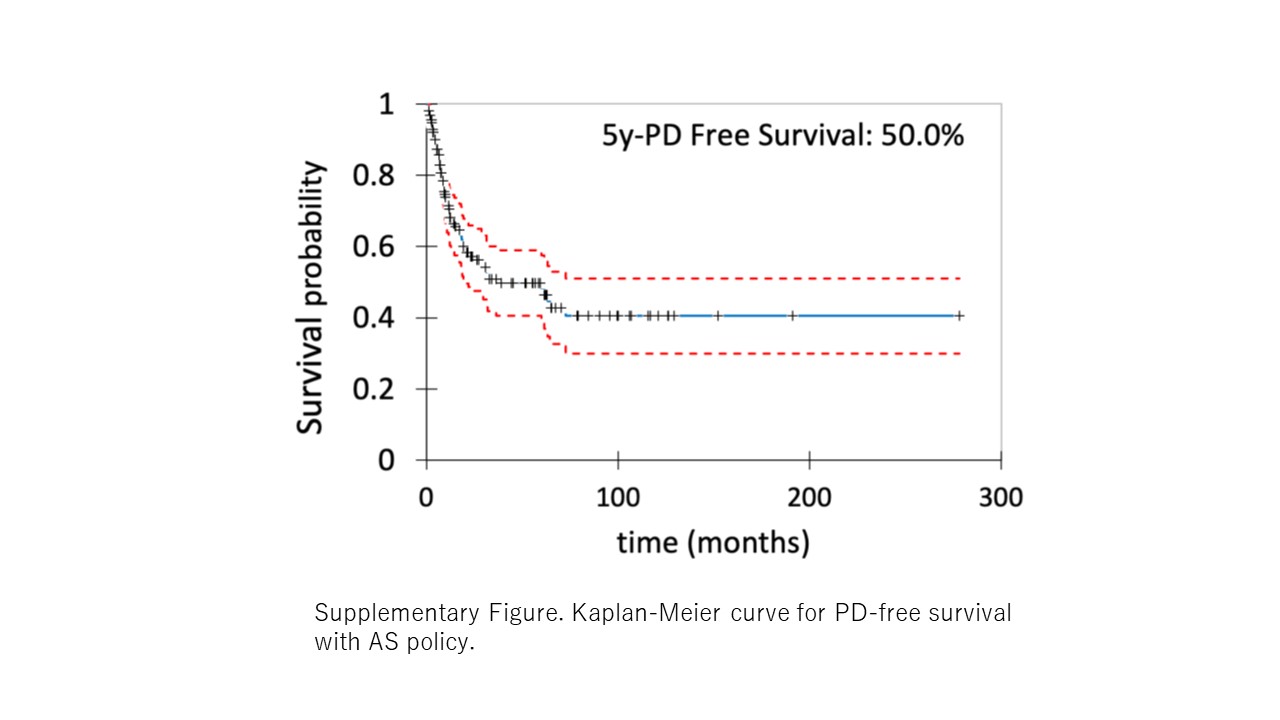

Supplement: Supplementary file 1 — Figure S1 [file CAM4-12-5245-s001.jpg]
