# Supplementary material for: Clinical results of active surveillance for extra‐abdominal desmoid‐type fibromatosis
Source: Cancer Med. 2022 Oct 9;12(5):5245–54. doi: 10.1002/cam4.5329 (PMC10028109; doi:10.1002/cam4.5329)
Supplement: Supplementary file 2 — Table S1 [file CAM4-12-5245-s002.docx]

| **Supplemental Table 1 Details of site of occurrence, CTNNB1 mutation and RECIST PD** | | | |
| --- | --- | --- | --- |
|  |  | Non-PD | PD |
| **Site** | Extremities | 22 | 25 |
|  | Abdominal wall | 24 | 17 |
|  | Other trunk | 30 | 15 |
|  | Retroperitoneal | 4 | 2 |
|  | Neck | 8 | 9 |
|  |  |  |  |
| ***CTNNB1* mutation** | T41A | 48 | 36 |
|  | T41I | 3 | 2 |
|  | S45F | 6 | 9 |
|  | S45P | 2 | 4 |
|  | H36P | 0 | 1 |
|  | Wild type | 21 | 11 |

PD; progressive disease
