# Supplementary material for: Clinical results of active surveillance for extra‐abdominal desmoid‐type fibromatosis
Source: Cancer Med. 2022 Oct 9;12(5):5245–54. doi: 10.1002/cam4.5329 (PMC10028109; doi:10.1002/cam4.5329)
Supplement: Supplementary file 3 — Table S2 [file CAM4-12-5245-s003.docx]

| **Supplemental Table 2 Details of site of occurrence, *CTNNB1* mutation and conversion to AT** | | | |
| --- | --- | --- | --- |
| Factor |  | Conversion to AT | Continue AS |
| **Site** | Extremities | 21 | 29 |
|  | Abdominal wall | 22 | 23 |
|  | Other trunk | 21 | 26 |
|  | Retroperitoneal | 2 | 4 |
|  | Neck | 8 | 12 |
|  |  |  |  |
| ***CTNNB1* mutation** | T41A | 39 | 52 |
|  | T41I | 2 | 3 |
|  | S45F | 13 | 4 |
|  | S45P | 2 | 4 |
|  | H36P | 1 | 0 |
|  | Wild type | 15 | 19 |

AT; active treatment, AS; active surveillance
